# Effect of Kelulut Honey on the Cellular Dynamics of TGFβ-Induced Epithelial to Mesenchymal Transition in Primary Human Keratinocytes

**DOI:** 10.3390/ijerph17093229

**Published:** 2020-05-06

**Authors:** Abid Nordin, Shiplu Roy Chowdhury, Aminuddin Bin Saim, Ruszymah Bt Hj Idrus

**Affiliations:** 1 Department of Physiology, Faculty of Medicine, Cheras, Kuala Lumpur 56000, Malaysia; m.abid.nordin@gmail.com; 2 Tissue Engineering Centre, Universiti Kebangsaan Malaysia Medical Centre, Cheras, Kuala Lumpur 56000, Malaysia; shiplu56@gmail.com; 3 Ear, Nose & Throat Consultant Clinic, Ampang Puteri Specialist Hospital, Ampang, Selangor 68000, Malaysia; aminuddin_saim@yahoo.com

**Keywords:** honey, cell morphology, scratch assay, keloid scar, migration, wound healing

## Abstract

Over-induction of epithelial to mesenchymal transition (EMT) by tumor growth factor beta (TGFβ) in keratinocytes is a key feature in keloid scar. The present work seeks to investigate the effect of Kelulut honey (KH) on TGFβ-induced EMT in human primary keratinocytes. Image analysis of the real time observation of TGFβ-induced keratinocytes revealed a faster wound closure and individual migration velocity compared to the untreated control. TGFβ-induced keratinocytes also have reduced circularity and display a classic EMT protein expression. Treatment of 0.0015% (*v*/*v*) KH reverses these effects. In untreated keratinocytes, KH resulted in slower initial wound closure and individual migration velocity, which sped up later on, resulting in greater wound closure at the final time point. KH treatment also led to greater directional migration compared to the control. KH treatment caused reduced circularity in keratinocytes but displayed a partial EMT protein expression. Taken together, the findings suggest the therapeutic potential of KH in preventing keloid scar by attenuating TGFβ-induced EMT.

## 1. Introduction

Over-induction of epithelial to mesenchymal transition (EMT) by transforming growth factor beta (TGFβ) in keratinocytes is a key feature in scarred wound and keloid formation [1]. Keloid is the formation of excessive fibrous tissue during the process of cutaneous wound healing. It can extend beyond the original wound borders, resulting in disfiguring scar [2]. In extreme cases, keloid scarring can cause significant morbidity due to decreased range of motion, pain, itching, and impaired self-image and quality of life [3].

EMT has been implicated as an important event during the re-epithelialization process of wound healing [4]. EMT allows immotile epithelial cells to assume motile mesenchymal-like cells phenotype to migrate over the wound bed. EMT key features are the loss of epithelial integrity and gain of mesenchymal cell motility. The phenotypic transition of the cells is typically marked by the reduction of molecular marker that represents the epithelial state such as E-cadherin, or the elevation of molecular marker that represents the mesenchymal state such as vimentin [5].

In recent years, efforts to describe the cell phenotypic state beyond the conventional molecular and genetic markers have started to gain attention [6]. Advancements in the imaging technology and image analysis methodology enable the quantification of morphological parameters such as wound closure rate, migration speed, and migration directionality. These parameters can be used to describe the step-wise transitional dynamic of the different cell state, instead of a singular endpoint achieved with destructive molecular evaluation [7]. This helps in establishing the conceptual framework required to understand the much more complex nature of the in vivo behavior of keratinocytes during the EMT process. Furthermore, availability of open access image analysis software such as ImageJ by the National Institute of Health US, may be a more cost-effective alternative in understanding the EMT process [8].

TGFβ has been known to be a potent inducer of EMT in many epithelial cells such as the airway epithelial and corneal epithelial cells [9,10]. Hanh et al (2016) had shown that keratinocytes derived from a keloid scar, exhibit similar behavior to the normal keratinocytes when TGFβ was inhibited. This suggests that the keloid scar formation is caused by the overexpression of TGFβ during the wound healing process. Upon further investigation of the markers of EMT, they concluded that the keloid-derived keratinocytes displayed features of a partial EMT [1].

In terms of therapeutic target for skin fibrosis, O’ Kane et al (2014) had previously shown that the inhibition of the SMAD signaling was able to reverse the EMT-activation of TGFβ-induced keratinocytes [11]. Applications of small molecule drug in keloid scar treatment might be costly and may result in adverse effects. Thus, application of natural product as an alternative might prove to be more cost-effective and safer.

Use of honey as wound treatment ailment can be dated back to ancient Egypt [12]. Reports regarding the efficacy of honey in the clinical setting can be found from as early as 1966 covering wide range of wound types [13]. At the cellular level, honey has been proven to benefit various cellular components of wound healing such as keratinocytes [14], fibroblasts [15], macrophages [16], and endothelial cells [17]. In keratinocytes, honey has been reported to modulate keratinocytes’ proliferation [18], migration [19], and it’s epithelial to mesenchymal transition [20].

In Malaysia, the stingless bee is a more common honey producer compared to their sting bee counterparts. Stingless bee can be found in the backyards of many villagers all around the country. The stingless bee is locally known as Kelulut. Previously, we have reported a positive influence of our Kelulut honey (KH) on dermal fibroblasts [21].

The current study has a twofold objective. First, we aim to define the cellular dynamics during EMT using quantitative morphological parameters following TGFβ-induction in human primary keratinocytes. Then, we seek to evaluate the effect of our KH on these cellular dynamics of the TGFβ-induced EMT in human primary keratinocytes.

## 2. Materials and Methods 

### 2.1. Ethical Consideration

This study was approved by the Universiti Kebangsaan Malaysia Research Ethics Committee (UKMREC) with approval code: FF-2017-008. Informed consent was obtained from the parents/guardian of patient undergoing circumcision procedure at Hospital Canselor Tunku Muhriz, Malaysia. The foreskin sample was collected and processed to obtain primary human keratinocytes.

### 2.2. Isolation of Primary Human Keratinocytes

Skin tissue was processed according to Manira et al. (2014) with a slight modification [22]. In brief, the skin was cleaned before cut into small pieces. The tissue matrices were then digested with 0.6% collagenase type I (Gibco, Gaithersburg, MD, USA) for 3–4 h. Next, 0.05% trypsin-EDTA (Gibco, USA) was used to dissociate the cell from the digested tissue. The cell suspension containing keratinocytes and fibroblasts cells of the skin were resuspended in co-culture medium (mixture of Nutrient Mixture F-12: Dulbecco’s Modified Eagle Medium (Sigma Aldrich, St. Louis, MO, USA) containing 10% FBS and EpiLife™ (Gibco, USA) at 1:1 ratio). The cells were then seeded into a 6-well culture plate (Nunc, Rochester, NY, USA) and incubated in 37 °C and 5% CO_2_ incubator. The co-culture medium was changed every 48 h until the co-culture reached approximately 90% confluency. Monoculture of keratinocytes were obtained through removal of fibroblasts by trypsinization with trypsin-EDTA for 3 min. The remaining keratinocytes were continued to be cultured with EpiLife™ medium in 37 °C and 5% CO_2_ incubator.

### 2.3. Scratch Wound Healing Assay

Human primary keratinocytes experiencing three passages were used in experiments and the seeding density was fixed at 10,000 cells/cm^2^ in a 12-well culture plate, which was determined by the trypan blue exclusion test on a hemocytometer. The cells were allowed to grow until confluence for 72 h with medium changed at 48 h. Upon confluency, scratch was introduced using a 10 µL pipette tips and the cells were washed with a warm Dulbecco phosphate buffer saline (DPBS). The cells were treated with EpiLife™ medium with or without TGFβ or KH. The 12-well culture plate was mounted on automated stage of the light microscope. Picture of the cell-free area were taken every 15 min for 24 h. The leading edges of the migrating cell population were manually tracked and quantified with validated image analysis software, ImageJ [23]. Percentage of wound area reduction was calculated where the wound area at 0 h is considered as 100%.

### 2.4. Cellular Dynamic Parameters

#### 2.4.1. Wound Closure Rate Analysis

The cell-free area within the confluent monolayer was measured using the image analyzer ImageJ. The cell-free area was traced manually along the wound edge and was calculated automatically by the software in µm^2^. The difference of the wound area reduction percentage at 5, 10, 15, and 20 h was measured and the rate was calculated as the percentage wound closure per hour.

#### 2.4.2. Migration Speed Analysis

The individual cell migration speed was determined using image analyzer ImageJ. At least 20 individual cells at the wound edge were traced and the x and y coordinate of each cell was measured automatically by the software in µm. The distance traveled by the migrating cells at 5, 10, 15, and 20 h were calculated by the formula

Distance = √((x_2_ − x_1_)^2^ + (y_2_ − y_1_)^2^)

The migration speed was then calculated as the distance (µm) traveled per hour.

#### 2.4.3. Migration Directionality Analysis

The directionality of cell migration was calculated by dividing the displacement of the migrating cell divided by the distance traveled for each time points. The average of the cell migration directionality at all time points was calculated.

### 2.5. Cell Circularity

Epidermal keratinocytes cells experiencing three passages were used in experiments and the seeding density was fixed at 10,000 cells/cm^2^ in 6-well culture plate, which was determined by the trypan blue exclusion assay on a hemocytometer. The cells were allowed to grow until 70% confluence for 48 h. The cells were treated with or without TGFβ or KH in the EpiLife™ medium. After 24 h, pictures of the cells were taken, and the resulted image was analyzed for cell circularity using the image analyzer ImageJ. At least 100 individual cells within the confluent monolayer were traced along the cell edge and the circularity index of each cell was measured automatically by the software. The remaining cells were washed with DPBS before being fixed with 4% paraformaldehyde overnight for immunocytochemical analysis.

### 2.6. Immunocytochemistry

The fixed cells were permeabilized for 20 min with 0.5% Triton X-100 solution (Sigma Aldrich, USA) and then blocked with 10% goat serum for 1 hour at 37 °C. The cells were then incubated with 1:200 mouse anti-E-cadherin antibody (ab1416) and 1:2000 rabbit anti-Vimentin antibody (ab92547) (Abcam, Eugene, OR, USA) overnight at 4 °C. On the following day, the cells were washed before being incubated with 1:300 diluted Alexa Fluor 594 anti-rabbit IgG (Invitrogen, Waltham, MA, USA) and Alexa Fluor 488 anti-mouse (Invitrogen, USA) for 1 hour at 37 °C. Nuclei were counterstained with DAPI [24]. Fluorescence images were captured with a Nikon Eclipse Ti fluorescence microscope (Nikon, Tokyo, Japan). The relative fluorescence intensity was calculated from five independent fields of images.

### 2.7. Honey Preparation

Honey of the stingless bee, Trigona itama, locally known as Kelulut was obtained from Ladang Kelulut Bimbingan MARDI, Selangor, Malaysia. Fresh honey was withdrawn using 20 mL syringe from the honey pot into a 50 mL unsterile glass container. The Kelulut honey (KH) sample was then diluted into equal volume of sterile ultra-pure water to get 50% volume/volume KH stock dilution. Appropriate KH dilutions were prepared prior to each individual experiment accordingly. For the dose-response evaluation, 1:2 serial dilutions of KH in EpiLife™ medium from the stock dilution were prepared (0.0015–25%; *v*/*v*).

### 2.8. MTT Cell Viability Assay

3-(4, 5-dimethylthiazolyl-2)-2, 5-diphenyltetrazolium bromide (MTT) is a yellow tetrazolium salt that will form intracellular purple formazan upon reduction by viable cells which can be solubilized and quantified via spectrophotometer [25]. The cells experiencing three passages were used in experiments and the seeding density was fixed at 10,000 cells/cm^2^ in 48-well culture plate, which was determined by the trypan blue exclusion assay on a hemocytometer. Cells were allowed to attach for 24 h.

Following cell attachment, a 1:2 serial dilutions of KH in EpiLife™ medium were prepared from the 50% stock, producing 25%, 12.5%, 6.25%, 3.125%, 1.56%, 0.78%, 0.39%, 0.192%, 0.096%, 0.048%, 0.024%, 0.012%, 0.006%, 0.003% and 0.0015% KH concentrations. Human epidermal keratinocytes viability was evaluated upon exposure of different concentrations of KH for 24 h. EpiLife™ medium without KH was used as an untreated control. Following 24 h exposure, cells were incubated with 10 µL MTT reagent diluted with 90 µL of EpiLife™ medium for 4 h. Viable cells lead to the formation of purple color precipitates inside the cell, which solubilized with 100 µL incubation of dimethyl sulfoxide (DMSO) for 2 h in the dark. Changes in color was measured using spectrophotometer.

To assess the long-term effect of different KH concentrations on human epidermal keratinocytes proliferation, the cell viability at 24, 72 and 120 h were monitored using MTT. Human epidermal keratinocytes were supplemented with selected KH concentrations (0.0015% and 1.56%). EpiLife™ medium without KH is used as an untreated control.

### 2.9. Statistical Analysis

All data were presented as mean ± standard deviation. The migration velocity distribution was presented as frequency distribution histogram. Statistical analysis was performed using GraphPad Prism (version 7.0; GraphPad Software, San Diego, CA, USA). Student t-test was used to compare the results of two groups. One-way analysis of variance (ANOVA) was used to compare the results of multiple groups. A *p*-value less than 0.05 was considered as significant.

## 3. Results

### 3.1. TGFβ Induction Enhance In Vitro Keratinocytes Wound Closure

**Figure 1:**
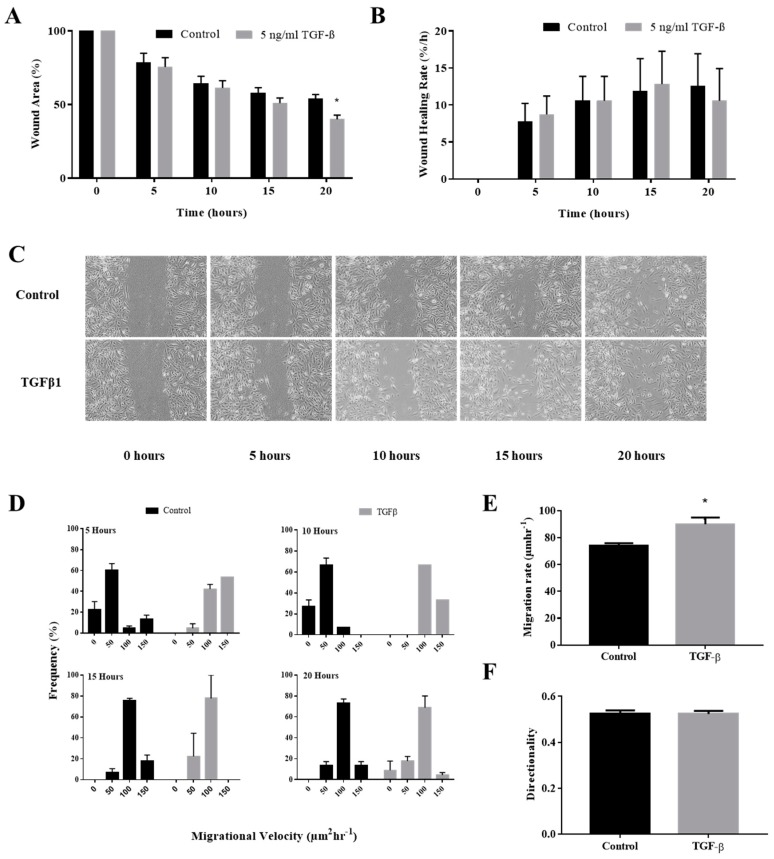
Effect of tumor growth factor beta (TGFβ) induction to migration behavior of keratinocytes. Cells cultured in 12-well plates were mechanically scratched with a sterile 10 µL pipette tip and then allowed to re-epithelialize for 24 h at 37 °C in the presence or absence of TGFβ. (**A**) The bar represents measurements of wound closure expressed as the percentage of initial wound area at 5, 10, 15, and 20 h. (**B**) The bar graph represents rate of wound healing derived from the wound area measurements at 5, 10, 15, and 20 h; (**C**) Representative images of scratch wound; (**D**) The histogram represents distribution of keratinocytes that migrates at 0–50, 50–100, 100–150, and <150 µm/h velocity at 5, 10, 15, and 20 h; (**E**) The average migration velocity of keratinocytes at all time points. (**F**) The average directionality of keratinocytes migration at all time points. The * indicate significant differences with *p* < 0.05 according to student t-test analysis.

Treatment of TGFβ resulted in enhanced wound closure at 5, 10, 15 and 20 h compared to the control. Complete closure of the wound was observed at 25 h. At 20 h, TGFβ treatment resulted in 40.0 ± 2.8% wound area compared to the 54.0 ± 2.8% wound area of the control. This difference was significant with a *p* value of 0.033. When the wound closure rate was calculated, no significant difference between the two treatments was observed at all time points. The wound area reduction and its calculated rate under TGFβ induction were depicted in Figure 1A,B respectively.

### 3.2. TGFβ Induction Increased Individual Keratinocytes Migration Velocity

Migration velocity of individual keratinocytes at the edge of the wound was analyzed to evaluate the migration behavior during EMT. Treatment of TGFβ caused the majority of keratinocytes to migrate at a velocity of <100 µm/h at 5, 10, 15, and 20 h. In contrast, majority of the control keratinocytes were still migrating at around 0–100 µm/h at 5 and 10 h before shifting to <100 µm/h velocity at 15 and 20 h. This shows that TGFβ enhances the keratinocytes migration at earlier time points compared to the control as shown in Figure 1D.

When average migration velocity was calculated, TGFβ-induced keratinocytes migrate at the velocity of 89.9 ± 5.00 µm/h compared to the velocity of 74.1 ± 1.89 µm/h for the control. This difference was significant with a p value of 0.0181. The average value of migration velocity with TGFβ induction were depicted in Figure 1E.

### 3.3. TGFβ Induction Did Not Affect the Directionality of the Keratinocytes Migration

Directional movement of individual keratinocytes at the edge of the wound was analyzed to evaluate the migration behavior during EMT. The average directionality index was 0.53 ± 0.01 and 0.52 ± 0.01 for the control and the TGFβ induction, respectively. No significant difference was observed in terms of the directionality index following TGFβ induction as depicted in Figure 1F.

### 3.4. TGFβ Induction Reduced the Circularity of the Keratinocytes

**Figure 2:**
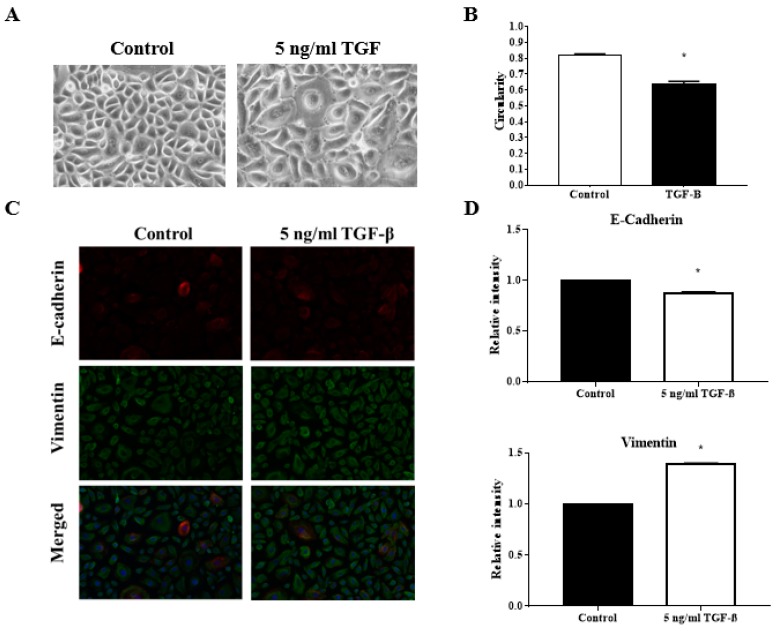
Effect of TGFβ induction to endpoint outcome of epithelial to mesenchymal transition (EMT). Cells cultured in 6-well plates in the presence or absence of TGFβ were analyzed for circularity parameters and EMT markers expression. (**A**) Representative images of keratinocytes cultured in the presence or absence of TGFβ; (**B**) Quantitative analysis of the circularity index of keratinocytes cultured in the presence or absence of TGFβ; (**C**) Representative fluorescence images of keratinocytes cultured in the presence or absence of TGFβ stained with antibodies against E-cadherin and vimentin; (**D**) Quantitative analysis of the relative intensity of E-cadherin and vimentin expression of keratinocytes cultured in the presence or absence of TGFβ. The * indicate significant differences with *p* < 0.0001 according to student t-test analysis.

The decreased in the circularity index of the keratinocytes in culture indicate the endpoint outcome of EMT occurrence. Indeed, when treated with TGFβ, a known inducer of EMT, reduction in keratinocytes circularity index can be observed. The average circularity index was reduced from 0.82 ± 0.01 in the control to 0.64 ± 0.02 with TGFβ induction. This difference was significant with a *p* value of <0.0001. Figure 2A shows the image of the keratinocytes under different treatments while Figure 2B described the analysis of the circularity index.

### 3.5. TGFβ Induction Exhibit Classical EMT Markers Expression

Treatment of TGFβ triggers the downregulation of E-cadherin and the upregulation of vimentin. Fluorescence intensity quantification revealed a reduction of relative intensity (RI) of E-cadherin expression to 0.88 ± 0.008 and increment of RI of Vimentin expression to 1.39 ± 0.004. Both differences were significant against the control with a *p* value of <0.0001. The fluorescence image of the protein expression was displayed in Figure 2C while Figure 2D described the analysis of fluorescence intensity.

### 3.6. KH Is Non-Toxic at Low Concentrations

**Figure 3:**
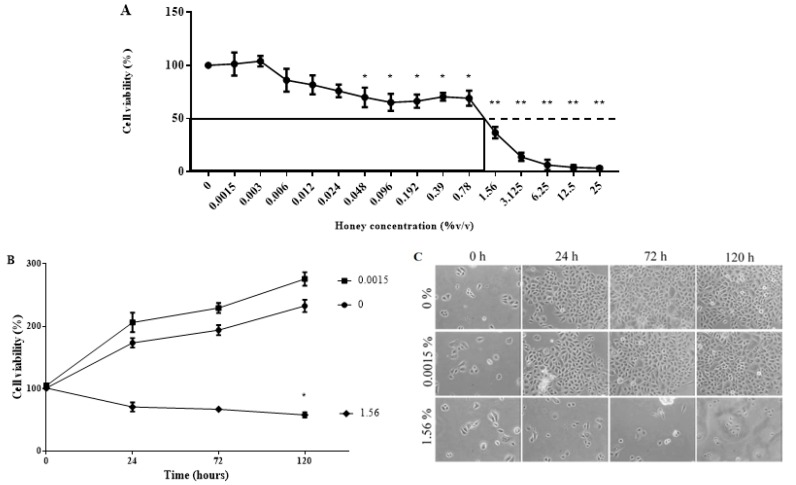
Effect of Kelulut honey (KH) on keratinocytes viability. The MTT assay was used to evaluate the viability of keratinocytes; (**A**) at 24 h with KH treatment at doses ranging from 0.0015–25% (*v*/*v*); and (**B**) at 24, 72 and 120 h with KH treatment at doses of 0, 0.0015, and 1.56% (*v*/*v*). (**C**) Representative images of keratinocytes cultured at 24, 72, and 120 h with KH doses of 0, 0.0015, and 1.56% (*v*/*v*). Significant differences according to student t-test analysis with *p* < 0.05 was indicated by * and *p* < 0.001 was indicated by **.

Treatment of Kelulut honey (KH) in epidermal keratinocytes demonstrated a dose-dependent effect on the cell viability at 24 h. At the lowest concentrations of KH (0.0015%), the cell viability is comparable to the control. The KH concentration at 0.048% starts to show a significant reduction of the viability at around 70%. KH caused 50% inhibition of cell viability at half maximal inhibitory concentration (IC_50_) of 1.56%. Figure 3A depicts the dose-response curve of KH and the epidermal keratinocyte cell viability.

Following the dose-response curve, the lowest concentration (0.0015%) and the IC_50_ concentration (1.56%) were chosen to evaluate the effect of KH on keratinocytes cell proliferation upon long-term exposure. Treatment with 0.0015% KH maintains the viability of the keratinocytes comparable to the untreated control. As expected, the IC_50_ concentration of KH negatively impacted the keratinocytes viability at all time points. The growth curve of epidermal keratinocytes at different time points following treatment of KH is shown in Figure 3B. Images of epidermal keratinocytes at different time points following treatment of KH is shown in Figure 3C.

### 3.7. KH Modulates TGFβ Induced Enhancement of In Vitro Keratinocytes Wound Healing

The lowest dose of KH (0.0015%) was evaluated in this study. Similar to the results in Figure 1A, TGFβ-induced keratinocytes demonstrated enhancement of wound healing compared to the control at 5, 10, 15 and 20 h. Complete closure of the wound was observed at 25 h. KH treatment reverses the enhancement of the keratinocytes’ wound closure by TGFβ induction, bringing it back to the level similar the control. At 20 h, co-treatment of KH and TGFβ resulted in significantly larger wound area of 52.6 ± 1.6%. This was similar to 53.9 ± 2.8% wound area in the control but significantly different to the 39.9 ± 2.9% wound area with TGFβ induction alone with a *p* value < 0.0001.

Treatment of KH alone exhibited similar wound healing trend as the TGFβ induction alone. However, although not significant, KH demonstrated a larger wound area when compared to the control at earlier time points, particularly at the 5 and 10 h, indicating slower healing. At 15 h, the wound area reduction starts to supersede the control before finally showing significant reduction at 20 h. The wound area at 20 h was 42.6 ± 1.7% in the KH group compared to 53.9 ± 2.8% in the control. This difference was significant with a *p* value of 0.0023.

**Figure 4:**
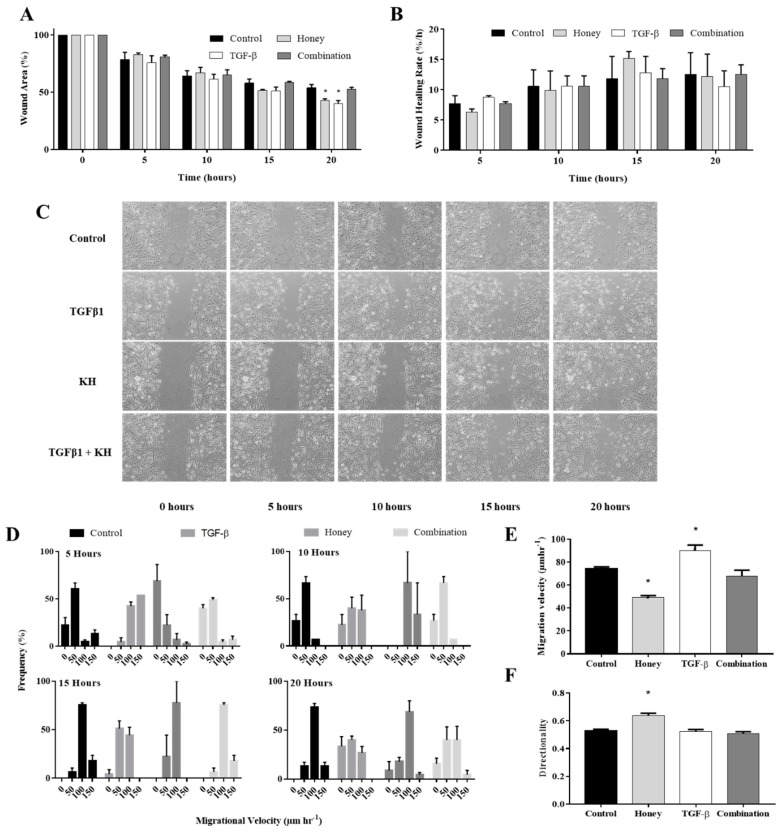
Effect of KH on the TGFβ-induced migration behavior of keratinocytes. Cells cultured in 12-well plates were mechanically scratched with a sterile 10 µL pipette tip and then allowed to re-epithelialize for 24 h at 37 °C with KH treatment in the presence or absence of TGFβ. Cells cultured without KH or TGFβ were analyzed as negative control. (**A**) The bar represents measurements of wound closure expressed as the percentage of initial wound area at 5, 10, 15, and 20 h. (**B**) The bar graph represents rate of wound healing derived from the wound area measurements at 5, 10, 15, and 20 h; (**C**) Representative images of scratch wound; (**D**) The histogram represents distribution of keratinocytes that migrates at 0–50, 50–100, 100–150 and <150 µm/h velocity at 5, 10, 15, and 20 h; (**E**) The average migration velocity of keratinocytes at all time points; (**F**) The average directionality of keratinocytes migration at all time points. The * indicate significant differences with *p* < 0.05 according to student t-test analysis.

Analysis of the wound closure rate did not show any significant differences between the treatments at all time points. The wound area reduction and its calculated rate for KH and TGFβ treatment were depicted in Figure 4A,B respectively.

### 3.8. KH Modulates TGFβ Induced Enhancement of Keratinocytes Migration Velocity

Treatment of KH reverses the enhanced migration velocity of the TGFβ induced keratinocytes to resembles the migration velocity of the control. Similar to the control, co-treatment of KH and TGFβ resulted in more keratinocytes to migrate at a velocity of 0–100 µm/h at 5 and 10 h before shifting to <100 µm/h velocity at 15 and 20 h. With the exception to the majority of keratinocytes migrating at 0–50 µm/h at 5 h, treatment of KH alone resulted in equal distribution of migration velocity at 10, 15 and 20 h. The distribution of keratinocytes migration velocity treated with KH and TGFβ is illustrated in Figure 4D.

In terms of average migration velocity, KH treatment reverses the increased velocity induced by TGFβ from 90.0 ± 5.00 µm/h to 67.9 ± 5.12 µm/h. This reduction was statistically significant with a *p* value of <0.0001. The average migration velocity of the KH treatment alone at 49.2 ± 1.68 µm/h was also revealed to be significantly lower than the 74.0 ± 1.88 µm/h of the control with a *p* value < 0.0001. Figure 4E summarizes the average migration velocity of keratinocytes under KH and TGFβ treatment.

### 3.9. KH alone Improves the Keratinocytes Migration Directionality

No significant difference was observed in terms of directionality when TGFβ induced keratinocytes were treated with KH. However, treatment of KH alone leads to a more directional migration compared to all treatment groups. Directionality index was 0.64 ± 0.02 with KH only treatment and this difference was statistically significant (<0.0001) compared to the 0.53 ± 0.01, 0.52 ± 0.01 and 0.50 ± 0.01 of the TGFβ induced, co-treatment of TGFβ and KH, and the control group, respectively. The directionality index of keratinocytes treated with KH and TGFβ is illustrated in Figure 4F.

### 3.10. KH Reverses TGFβ Induced Reduction of the Keratinocytes Circularity

**Figure 5:**
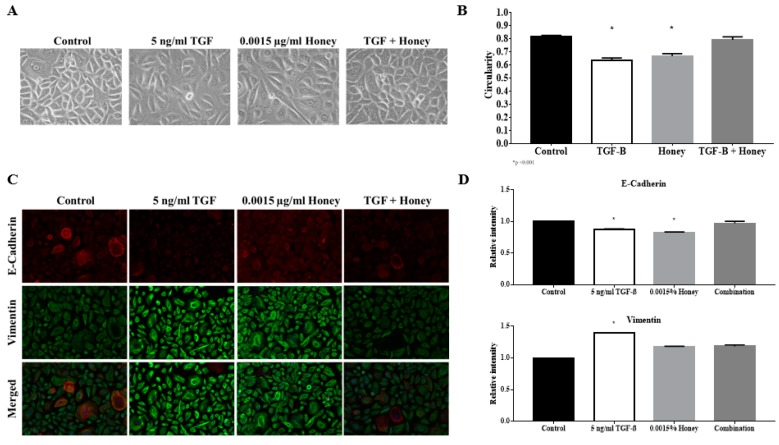
Effect of KH on TGFβ-induced EMT. Cells cultured in 6-well plates treated with KH in the presence or absence of TGFβ were analyzed for circularity parameters and EMT markers expression. Cells cultured without KH or TGFβ were analyzed as a negative control. (**A**) Representative images of keratinocytes cultured in different conditions; (**B**) Quantitative analysis of the circularity index of keratinocytes cultured in different conditions; (**C**) Representative fluorescence images of keratinocytes cultured in different conditions stained with antibodies against E-cadherin and vimentin; (**D**) Quantitative analysis of the relative intensity of E-cadherin and vimentin expression of keratinocytes cultured in different conditions. The * indicate significant differences with *p* < 0.0001 according to student t-test analysis.

Treatment of KH reverses TGFβ induced keratinocytes’ circularity from 0.65 ± 0.02 to 0.79 ± 0.02, which was comparable to 0.80 ± 0.01 in the control. Interestingly, KH alone reduces keratinocytes’ circularity to 0.67 ± 0.02, which was comparable to the TGFβ induction. The image of epidermal keratinocytes and their circularity index under KH and TGFβ treatment were visualized in Figure 5A,B.

### 3.11. KH Reverses TGFβ Induced EMT Protein Expression

Treatment of KH reverses TGFβ induced EMT protein expression to the level comparable to the control. TGFβ induction reduces the E-cadherin expression to 0.87 ± 0.008 RI while treatment of KH restores the expression back to 0.97 ± 0.032 RI. In terms of vimentin, TGFβ induced expression of 1.40 ± 0.004 RI was suppressed by KH to 1.19 ± 0.021 RI, a level comparable to the control.

Treatment of KH alone exhibited partial EMT features where E-cadherin was downregulated but vimentin was not upregulated. The downregulation of E-cadherin was similar to TGFβ induction at 0.83 ± 0.008 RI. Unlike TGFβ induction, vimentin expression with treatment of KH alone was comparable to the control at 1.18 ± 0.005 RI. Fluorescence image of the immunostained cells under KH and TGFβ treatment, as well as the accompanying graph of fluorescence intensity. were displayed in Figure 5C,D, respectively.

## 4. Discussion

The current study revealed enhancement of the colonization of wound defects by keratinocytes, and their migration velocity by TGFβ induction. Alternatively, TGFβ induction also caused diminution of cell circularity into a mesenchymal-like spindle shape. When protein expression of the epithelial marker E-cadherin and mesenchymal marker vimentin was evaluated, TGFβ induction resulted in the classical EMT indicator of E-cadherin downregulation and vimentin upregulation. Our results suggest the validity of the quantitative morphological parameters to be used as indicator of EMT activation in human primary keratinocytes.

Enhancement of the keratinocytes wound closure in vitro by TGFβ induction has been reported previously. Hanh et al. (2013) induced normal keratinocytes with TGFβ to mimic the keratinocytes derived from keloid scar. Indeed, TGFβ induction caused the keratinocytes to colonize the wound defect faster than their untreated counterparts [3]. Absence of TGFβ gene has also been implicated with non-healing wounds. Pastar et al. (2010) found that the TGFβ signaling is functionally blocked in non-healing venous ulcer [26]. This highlights the importance of the balance in TGFβ signaling to achieve a complete and proper wound healing.

KH treatment was able to reverse the TGFβ induced wound closure enhancement. This suggests the therapeutic potential of KH to prevent over-induction of wound healing. Over-induction of wound healing is a characteristic of keloid scar [1]. Hence, KH has the potential to be used as anti-keloid agent. In untreated keratinocytes, KH seems to slow down the wound closure in the earlier time points. However, this effect was not significant and could be due by the variation of the initial wound size.

Treatment of 5 to 20 ng/mL TGFβ demonstrated dose-dependent effect on the magnitude of EMT in respiratory epithelial cell [24]. Similar effect was observed during the optimization stage of the current study (Data not shown). Since EMT is a reversible phenomenon, the reversal could be caused by the smaller magnitude of effect exerted by the 5 ng/mL TGFβ. A higher concentration of TGFβ could result in more persistent EMT and renders KH unable to reverse its effect.

A longstanding hypothesis of EMT in the context of wound healing is that it allows immotile epithelial cells to assume motile mesenchymal-like cells phenotype, to migrate over the wound bed [4]. Thus, increase in motility following TGFβ induction is expected. The increased migration velocity induced by TGFβ confirms the wound closure result. When keratinocytes migrate faster, they can colonize the wound defect faster. However, the increased migration velocity may indicate the over-induction of wound healing, a feature of keloid scarring [1]. As such, increased keratinocytes migration velocity can be an indicator of keloid wound healing.

KH treatment was able to reverse the TGFβ induced increase of migration velocity. This finding supports the aforementioned therapeutic potential of KH as an anti-keloid agent. In untreated keratinocytes, KH seems to slow down the migration velocity only in the earliest time points. This pattern of initial slow migration followed by a subsequent faster migration was in accordance to the wound closure result with KH in untreated keratinocytes.

One of the features of EMT is the loss of apical-basal polarity of the cells. This caused the circularity of the cell to reduce when observed under light microscope. The decreased in the circularity index of the keratinocytes in culture indicate the endpoint outcome of EMT occurrence. Circularity parameters have been included in reports of the EMT study in the neoplasm context [6,9]. However, it has not been seen in any EMT study in the wound healing context. In this study, we demonstrated the reliability of circularity parameters as an indicator of EMT activation.

Similar to what was reported by Hanh et al. (2016) and O’Kane et al. (2014), we were able to demonstrate the hallmark reduction of E-cadherin and elevation of vimentin with TGFβ induction in keratinocytes [1,11]. Most of the EMT study, include a wide array of molecular markers to evaluate EMT occurrence. In this study, only two markers were used and this might affect the interpretation of the findings.

At the transcriptional level, E-cadherin production is inhibited by the transcriptional factors Snail and Slug. Simultaneously, elevation of Snail and Slug also resulted in elevation of vimentin [27]. The partial EMT exhibited by KH suggested that it modulates the process circumventing the Snail and Slug transcription factors. However, it could also mean that KH might still induce Snail and Slug but prevents elevation of vimentin in other pathways. This can be answered by measuring the level of Snail and Slug expression.

In addition to Snail and Slug, EMT is also regulated by basic helix-loop-helix (bHLH) and zinc-finger E-box-binding (ZEB) transcription factors. Multiple signaling pathways cooperate in the initiation and progression of EMT including Smad, Rho-like GTPases, phosphoinositide 3-kinase (PI3K), and mitogen-activated kinase (MAPK) [28]. One of the advantages of in vitro cellular model such as the one in the current study is the ability to induce and inhibit specific signaling molecules to investigate its role in the action reported. Elucidating the molecular mechanism underlying the KH effect on EMT can be of great interest.

Honey has been implicated with the EMT process of wound healing. Upon monitoring the gene expression during re-epithelialization of keratinocytes, Ranzato et al. (2012) revealed that the treatment of honey, upregulated both the E-cadherin and vimentin expression [20]. This suggests that honey enhances keratinocyte migration while simultaneously maintaining epithelial integrity. In this study, KH treatment downregulated the E-cadherin but did not affect vimentin expression. We hypothesized that our keratinocytes lose their epithelial integrity without any cytoskeletal changes that may over-enhance their migration. This hypothesis was supported by the observation of initial slower migration, which sped up later on resulting in greater wound closure at the later time point. Taken together, the results revealed a differential effect of honey in a different context of the cell phenotypic state.

Directionality of the cell migration can play an important role in wound healing. A cell that moves at a greater distance might not necessarily be moving efficiently; either through taking larger and more variable steps, or not migrating in a straight path [29]. Factors that influence EMT such as the absence of Ovol gene, have been reported to affect directionality of cell migration [30]. However, there was no difference in terms of directionality between TGFβ induced keratinocytes and its untreated counterparts observed in this study. The greater directional migration observed in KH treatment in untreated keratinocytes, could be the result of partial modulation of the EMT by KH. In the control, cytoskeletal changes indicated by the upregulation of vimentin, may cause the individual keratinocytes to move more randomly compare to the unchanged cytoskeleton in KH treated keratinocytes.

The current study reported the safety of KH at low dosage. Inhibitory concentration at half maximal survival, or known as IC_50_, is a method employed to determine the toxic dose of a particular drug after a short exposure [31]. Among the safety dose of honey, we chose the lowest dose in our study, 0.0015%, to evaluate the effect of honey on other parameters of wound healing. The lowest dose was chosen to consider the cost-effectiveness of the final therapeutic product developed in the future.

In terms of the dose response viability, a study using Gelam honey and corneal keratocytes reported the same findings as the current study [32]. Glucose constitutes 30% of the stingless bee honey mass [33]. In cell metabolism, glucose acts a major source of energy. In the event of its excess, accumulation of oxidative stress from glucose auto-oxidation, non-enzymatic glycation, the formation of advanced glycation end products, and the overproduction of reactive oxygen species by mitochondria can occur [34]. These result in cytotoxicity instead of maintenance of the cell metabolism.

Furthermore, the acidity of honey might also influence cell viability in culture [33]. A balanced and stable pH, between 7.2 to 7.4 are ideal for nearly all cells in culture [35]. Hence, a disruption in the cell culture media pH can impede the cell viability and growth. However, addition of honey into the cell culture medium did not change the pH of the culture medium (data not shown). This could be due to the buffer included in the formulation of the culture media.

Long-term exposure of the keratinocytes cells with KH reinforced the results of the dose response study. KH at 0.0015% was able to maintain cell viability over time comparable to the control. As expected, the IC50 dose, 1.56% honey, resulted in the inhibition of the keratinocyte proliferation.

## 5. Conclusions

In conclusion, the current study established the reliability of quantitative morphological parameters in describing the cellular dynamics during EMT. More importantly, the cellular phenotypic transition during EMT can be attenuated by KH. Taken together, the findings demonstrated the potential of KH to be used in scar-less wound healing application. This preliminary in vitro work should be supplemented with pre-clinical and clinical study in hypertrophic or keloid scar.

## Abbreviations

ANOVA	One-way analysis of variance
bHLH	Basic helix-loop-helix
DMSO	Dimethyl sulfoxide
EMT	Epithelial to mesenchymal transition
IC_50_	Half maximal inhibitory concentration
KH	Kelulut honey
MAPK	Mitogen-activated kinase
MTT	3-(4, 5-dimethylthiazolyl-2)-2, 5-diphenyltetrazolium bromide
PI3K	Phosphoinositide 3-kinase
RI	Relative intensity
TGFβ	Tumor growth factor beta
UKMREC	Universiti Kebangsaan Malaysia Research Ethics Committee
ZEB	Zinc-finger E-box-binding
